# Identification of network topological units coordinating the global expression response to glucose in *Bacillus subtilis *and its comparison to *Escherichia coli*

**DOI:** 10.1186/1471-2180-9-176

**Published:** 2009-08-24

**Authors:** Carlos Daniel Vázquez, Julio A Freyre-González, Guillermo Gosset, José Antonio Loza, Rosa María Gutiérrez-Ríos

**Affiliations:** 1 Departamentos de Microbiología Molecular, Instituto de Biotecnología, Universidad Nacional Autónoma de México, Apdo. Postal 510-3, Cuernavaca, Morelos 62250, México; 2 Ingeniería Celular y Biocatálisis, Instituto de Biotecnología, Universidad Nacional Autónoma de México, Apdo. Postal 510-3, Cuernavaca, Morelos 62250, México; 3 Instituto de Ciencias Nucleares, Universidad Nacional Autónoma de México Apartado Postal 70-543, 04510 México D.F., México

## Abstract

**Background:**

Glucose is the preferred carbon and energy source for *Bacillus subtilis *and *Escherichia coli*. A complex regulatory network coordinates gene expression, transport and enzymatic activities, in response to the presence of this sugar. We present a comparison of the cellular response to glucose in these two model organisms, using an approach combining global transcriptome and regulatory network analyses.

**Results:**

Transcriptome data from strains grown in Luria-Bertani medium (LB) or LB+glucose (LB+G) were analyzed, in order to identify differentially transcribed genes in *B. subtilis*. We detected 503 genes in *B. subtilis *that change their relative transcript levels in the presence of glucose. A similar previous study identified 380 genes in *E. coli*, which respond to glucose. Catabolic repression was detected in the case of transport and metabolic interconversion activities for both bacteria in LB+G. We detected an increased capacity for *de novo *synthesis of nucleotides, amino acids and proteins. A comparison between orthologous genes revealed that global regulatory functions such as transcription, translation, replication and genes relating to the central carbon metabolism, presented similar changes in their levels of expression. An analysis of the regulatory network of a subset of genes in both organisms revealed that the set of regulatory proteins responsible for similar physiological responses observed in the transcriptome analysis are not orthologous. An example of this observation is that of transcription factors mediating catabolic repression for most of the genes that displayed reduced transcript levels in the case of both organisms. In terms of topological functional units in both these bacteria, we found interconnected modules that cluster together genes relating to heat shock, respiratory functions, carbon and peroxide metabolism. Interestingly, *B. subtilis *functions not found in *E. coli*, such as sporulation and competence were shown to be interconnected, forming modules subject to catabolic repression at the level of transcription.

**Conclusion:**

Our results demonstrate that the response to glucose is partially conserved in model organisms *E. coli *and *B. subtilis*, including genes encoding basic functions such as transcription, translation, replication and genes involved in the central carbon metabolism.

## Background

During the last decades, an increase in the quantity of available data referring to biological systems has enabled the development of new paradigms and methods for their analysis, with the purpose of formulating coherent opinions regarding cellular events, both locally and globally. Recently, a network based approach for the representation of cellular component interactions has proven highly successful, when applied to the study of genetic expression regulation and the mechanics of cellular metabolism [1]. This approach permits the identification of the effects caused by interactions among proteins and other cellular components; thus for the first time presenting the possibility of visualizing the cell as a system. In the light of the successful results obtained when applying this approach to the model organism *Escherichia coli *[2]; this type of analysis is now being applied to other organisms such as the soil bacterium *Bacillus subtilis *[3].

For many decades *B. subtilis *has represented the most important model for the study of firmicutes. Its genome includes 4106 predicted genes, with a G+C content of 43.5%. Currently, the functions of about half of the predicted genes are known. At the time when *E. coli *became the most important bacterial model, the study of *B. subtilis *was initiated, partly due to its relative facility for genetic manipulation, but also in large part due to its capacity to form spores [4,5]. Currently, *B. subtilis *continues to be employed as an important biological model, especially for a large number of studies related to genetic regulation and metabolism. Furthermore, *B. subtilis *is an organism which attracts considerable commercial interest, as for many years it has been used as an industrial producer of enzymes and metabolites.

*B. subtilis *is a free living bacterium and therefore, it must adapt to changes in its environment, for example nutrient availability or fluctuations in temperature. Among nutrients, sugars and other carbon sources are particularly important, as these usually also provide the cell with metabolic energy. Microbes are constantly sensing the levels and types of carbon sources present in the environment. This function is carried out in most bacteria, including *B. subtilis*, by the phosphoenolpyruvate: sugar phosphotransferase system (PTS) [6]. The PTS is a protein system composed of general and sugar-specific components. The enzyme I (EI) and the phosphohistidine carrier protein (HPr), relay a phosphoryl group from phosphoenolpyruvate (PEP) to the sugar-specific proteins IIA and IIB. The last component of this system, IIC (in some cases also IID), is an integral membrane protein permease that recognizes and transports the sugar molecules, which are phosphorylated by component IIB. There are several PTS component II encoded in the genome of *B. subtilis*, each one having a specific sugar as substrate [7].

*B. subtilis *displays a pattern of preferential carbon source consumption, depending on their varying metabolic rates, which in turn result in differing growth rates. Glucose is considered the preferred carbon source as it sustains the highest growth rate and the same applies in the case of *E. coli *[7]. Repression of the genes involved in the metabolism of sugars is part of a global phenomenon known as carbon catabolite repression (CCR). In *B. subtilis*, this phenomenon occurs due to PTS-mediated phosphorylation of regulatory proteins and GlcT controlling antitermination. In most cases, CCR is defined by the presence of catabolic responsive elements sites (CRE) in the 5' regions of the regulated genes. The CRE DNA sequences are recognized by the catabolite control protein A (CcpA), whose repressed gene encoding functions relate to the utilization of alternative carbon sources and other stress conditions, in the presence of a preferential carbon source, such as glucose [8,9].

A global view of the cellular transcriptional response can now be accomplished using microarray technology. This type of of study provides an instantaneous snapshot of the way cells function, under specific conditions. The data generated using this technology is useful for revealing the nature of the complex regulatory interactions in the cell. At the present time several reports exist, describing the use of microarrays to study *B. subtilis *under diverse conditions; for example in the presence of acid [10], in response to thermic shock [11], anaerobiosis [12] and in the presence or absence of glucose [8], among others. These results provide data that will enable the construction of a detailed regulatory network and help to elucidate how regulatory proteins interact with their effectors.

In this work, we analysed the regulatory network of *B. subtilis*, when grown in a complex medium in the absence or presence of glucose. This study enabled the identification of network modules, coordinating the response of genes with related functions. The results obtained were compared to those from our previous study where *E. coli *was employed[13].

## Results

### Global transcriptome response to the presence of glucose in complex medium, in *Bacillus subtilis*

**Figure 1:**
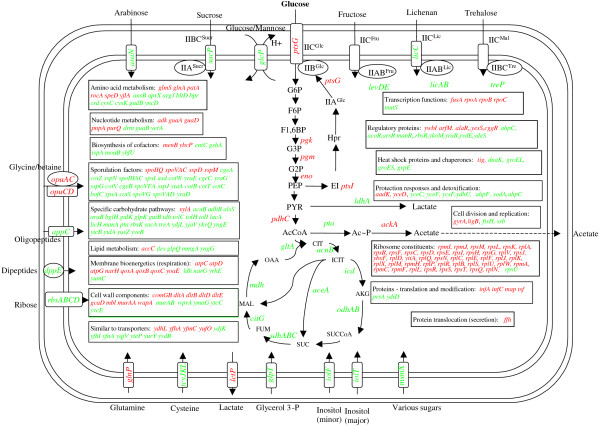
**A metabolic view of the transcriptome profile of *B. subtilis*, comparing growth in LB+G to that in LB**. Genes displaying higher and lower transcript levels, due to the presence of glucose are shown in red and green respectively. Abbreviations: AcCoA, acetyl coenzyme-A; Ac~P, acetyl phosphate; AKG, α-ketoglutarate; CIT, citrate; F1,6BP, fructose-1,6-bisphosphate; F6P, fructose-6-phosphate; FUM, fumarate; G3P, glycerol-3-phosphate; G6P, glucose-6-phosphate; ICIT, isocitrate; MAL, malate;OAA, oxaloacetate; PEP, phosphoenolpyruvate; PYR, pyruvate; SUC, succinate; SUCCoA, succinyl-CoA;. G2P 2-phospho-glycerate.

We performed an analysis of transcriptome data obtained from previous reports of experiments, employing *B. subtilis *[8]. Following the procedure described in the methods section, 504 genes were found to display significant differential expression, when grown in either the absence or presence of glucose and these were compared (see Additional File 1: Table 1SM). In figure 1, we present the genes with known functions, where transcription was found to consist of a response to the presence of glucose in LB medium (LB+G). Among this set of genes, we found those induced in the presence of glucose, to be related to transport and metabolism, for example the general PTS protein enzyme I and the glucose-specific IICB^Glc ^permease, as well as the *pgk*, *pgm*, *eno *and *pdhC *genes, which encode enzymes from the glycolytic pathway. The transcriptional activation of the aforementioned genes is expected to increase the cellular glucose capacity for transport and catabolism. On the other hand, down-regulation was observed in the case of genes encoding most of the enzymes from the TCA cycle and the glyoxylate bypass [7].

A clear glucose-dependent repressive effect was observed for genes encoding transporters, periplasmic receptor proteins and enzymes related to the import and catabolism of alternative carbon and nitrogen sources; for example carbohydrates, amino acids, lactate, glycerol 3-P, oligopeptides, dipeptides and inositol [7]. This transcriptome pattern is the expected result of CCR, exerted by glucose. Interestingly, we detected a general trend towards down-regulation in LB+G medium, in the case of genes encoding heat shock proteins and chaperones. This response suggests a higher stress condition and a higher protein turnover rate among cells growing in medium, which lacked glucose. Contrastingly, the presence of glucose caused an increase in the transcript level for genes encoding ribosome constituents. This response is consistent with the improved growth conditions provided, with the presence of glucose.

We also detected, lower transcript levels in the presence of glucose for gene encoding proteins involved in sporulation. This included regulatory proteins, enzymes and structural proteins involved in spore formation. This response is to be expected, in the light of the repressive effect that glucose exerts on the sporulation process [14].

### Topological analysis of a sub-network of *Bacillus subtilis*, responding to glucose

Data from DBTBS [15] was used to generate the known regulatory network of *B. subtilis*. The resulting network is composed of 1453 nodes and 2337 edges, showing an average clustering coefficient of 0.47. The degree distribution follows a power law, P(*k*) ~*k*^-2.0043^. These results are characteristic of a scale-free network, and strongly suggest the existence of a modular hierarchical organization. These properties are common to other previously described biological networks [1].

As described in the methods section, we selected a set of 504 genes shown to respond under the test conditions, with a significant level of expression. From this set, those genes not having a regulatory relation were eliminated from the regulatory network. The resulting network will be called the sub-network that responds to the presence of glucose. In this sub-network, 264 genes have known regulatory information, including sigma and transcription factors; TFs. As the sigma factor A is predominantly connected to almost every gene in the network, we decided to remove it from the subnetwork. Therefore, the final subnetwork used for further analysis includes 186 genes, 68 (TF) and 10 sigma factors.

**Figure 2:**
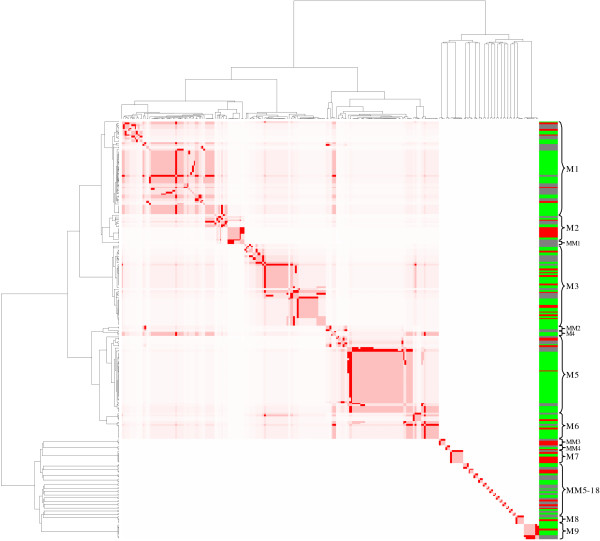
**Clustering results from the *B. subtilis *sub-network that responds to glucose**. The image shows the modular structures obtained using the clustering method. The figure is composed of a giant component with six modules (M1-6) and two mini-modules (MM1-2). Disconnected from the giant component, we have 16 mini-modules (MM3-18) and two modules (M8-9). The column on the right hand side shows the transcriptional response for each gene, according to the microarray data. Red color represents an increase in transcript level, green color represents a decrease in transcript level and grey color indicates no significant change in transcript level.

By applying a hierarchical agglomerative clustering algorithm to the sub-network, it was possible to group the transcription factors and the genes responding to glucose into topological modules (figure 2). The clustering algorithm grouped the genes in a giant component, composed of 6 modules which include members with more that one operon and two mini-modules (basically complex and simple regulons [16]). Additionally, disconnected from the giant component we discovered 16 mini-modules and 3 modules.

#### Carbon metabolism and stress response (M1)

The first module identified using this method, includes 39 genes distributed within two sub-modules: The first sub-module, includes 8 genes, belonging to two of the functional classes described in the SubtiList database [17]. In this submodule, 3 clustered genes related to anaerobic conditions are induced in the microarray data, table 1. This behavior appears to be consistent with observations from previous reports, indicating that the regulation of this gene regulatory cascade by an unknown sensor via ResDE, Fnr, and ArfM manifests differing growth, especially when both glucose and pyruvate are provided, or when glucose and mixtures of amino acids are present [18]. The other five genes included in this sub-module are encoding proteins, related to the heat shock response. These genes are repressed by the protein HrcA, which is auto-regulated and whose transcription can also be activated by ArfM. The microarray data indicate that the gene *arfM *is induced by glucose, suggesting that the protein ArfM activates transcription of *hrcA *and the encoded protein, whereas it represses *dnaK*, *grpE*, *groEL *and *groES*. The second sub-module includes 31 genes with a detected transcript level, 29 of which were repressed and 2 of which were induced. Out of this set, 30 of these are regulated by CcpA (catabolic control protein). These genes encode functions associated with the transport and degradation of alternative carbon sources.

**Table 1:** Modules and sub-modules found in the *B. subtilis *glucose-responding regulatory network.

Module	Physiological function	Genes
M1	Heat shock response	*acoR*(↓), *alsS*(↑), *arfM*, *alsR*, *ydiH*, *cydC*(↑), *dnaK*(↓), *grpE*(↓), *lctP*(↑), *hrcA*, *resD*, *groEL*(↓), *groES*(↓)
	
	Carbon catabolism	*glpK*(↓), *glpP*, *ahrC*, *rocR*, *iolR*, *araB*(↓), *araN*(↓), *acuB*(↓), *galK*(↓), *msmX*(↓), *pta*(↓), *bglH*(↓), *bglP*(↓), *yxiE*(↓), *licA*(↓), *licB*(↓), *licC*(↓), *licH*(↓), *treA*(↓), *treP*(↓), *ccpA*, *iolC*(↓), *iolF*(↓), *iolH*(↓), *iolI*(↓), *ccpB*, *xylR*, *xylA*(↑), *araR*, *treR*, *licR*, *licT*, *levD*(↓), *levE*(↓), *levR*, *sigL*, *rocA*(↑), *ydjK*(↓), *rbsA*(↓), *rbsB*(↓), *rbsC*(↓), *rbsD*(↓), *rbsK*(↓), *rbsR*(↓)

M2	Endospore formation	*citG*(↓), *codY*, *dppE*(↓), *hag*(↑), *abrB*, *sigH*, *spoVG*(↓), *yxnB*(↓)
	
	Sporulation	*dltA*(↑), *dltB*(↑), *dltD*(↑), *dltE*(↑), *mcpB*(↑), *yjcP*(↑), *yvyC*(↓), *sigD*

MM1	Sporulation	*sigX*, *spo0A*

M3	Prespore formation	*comA*, *yvrH*, *wprA*(↓), *degQ*(↓), *wapA*(↑), *sacA*(↓), *sacP*(↓), *degU*, *sacT*, *sacY*, *tenA*, *yveB*(↓), *sigG*, *gerKA*(↓), *ybxH*(↓), *sspD*(↑), *spoVAD*(↓), *spoVAC*(↑), *sspJ*(↓), *sspM*(↑), *adhB*(↓), *yraG*(↓), *yraE*(↓), *yraD*(↓), *yndE*(↑), *ylaJ*(↓), *sspN*(↓)
	
	Spore formation	*ctsR*, *bofC*(↓), *csbX*(↑), *sigF*, *spoVT*, *sigB*, *clpP*(↓), *rsbW*(↓), *ydaE*(↓), *ydhK*(↓), *yjgB*(↑), *gcaD*(↑), *yycD*(↓), *ysnF*(↓), *ypuB*(↑), *yoxB*(↓), *yotK*(↑), *yqhQ*(↓), *spoIIQ*(↑), *yfhD*(↓), *yfhE*(↓), *yhcM*(↓), *yqzG*(↓)

MM2	Glycerophospholipid metabolism	*glpQ*(↓), *glpT*(↓), *phoP*

M4	Hexuronate metabolism	*exuR*, *mmgA*(↓), *yjmC*(↓)

M5	Nitrogen metabolism	*glnR*, *glnA*(↑), *glnP*(↑), *kipR*, *tnrA*, *ykoL*(↓), *ykzB*(↑)
	
	Spore coat formation	*gerE*, *spoIIIC*, *spoIVCB*, *cotB*(↓), *cotC*(↓), *cotV*(↓), *cotW*(↓), *cotT*(↓), *cgeA*(↓), *cgeB*(↓), *cotZ*(↓), *sspG*(↓), *cgeC*(↓), *yurS*(↓), *yoaN*(↑), *yjcB*(↓), *spoVFA*(↓), *yisZ*(↓), *ykvP*(↓), *ykvQ*(↓), *ylbD*(↓), *ylbE*(↓), *cotS*(↓), *ywrJ*(↓), *ytxO*(↓), *ytcC*(↓), *ytaA*(↓), *yqfQ*(↓), *yodH*(↓), *yngK*(↓), *ymaG*(↓), *spsA*(↓), *spsI*(↓), *ycsF*(↓), *spoIIID*, *ylbO*, *yhcO*(↓), *yhcP*(↓), *ypqA*(↓), *ysnD*(↓)

M6	SOS response	*lexA*, *ybaK*(↓), *aprX*(↓), *yozM*(↓), *yozL*(↑)
	
	Prospore formation	*spoIIIAC*(↓), *yqfZ*(↓), *sigE*, *usd*(↓), *mbl*(↑), *yheD*(↓), *yjcA*(↓), *yncD*(↓), *yngE*(↓), *yngG*(↓), *ywdL*(↓)

MM3	Glycolysis	*cggR*, *eno*(↑), *pgk*(↑), *pgm*(↑)

MM4	Nitrogen assimilation	*fnr*, *narG*(↓), *narH*(↑)

M7	Competence	*comK*, *comGB*(↑), *cspB*(↓), *yhjC*(↓), *yhcD*(↑), *ssb*(↑), *rpsF*(↑), *rpsR*(↑)

MM5	Peroxide stress	*ahpC*(↓), *ahpF*(↓), *perR*

MM6	PTS-glucose system	*glcT*, *ptsG*(↑), *ptsI*(↑)

MM7	Amine and polyamine degradation	*bltD*(↓), *bltR*, *mta*

MM8	Extracytoplasmic	*sigY*, *yxlC*(↓), *yxlE*(↓)

MM9	Aspartate catabolism	*ansB*(↓), *ansR*

MM10	N/A	*lmrA*, *yxaH*(↓)

MM11	N/A	*fur*, *ydhU*(↓)

MM12	N/A	*rok*, *yydH*(↓)

MM13	Sorbitol catabolism	*gutB*(↓), *gutR*

MM14	Purine metabolism	*purQ*(↑), *purr*

MM15	N/A	*birA*, *ytbQ*(↑)

MM16	N/A	*yufM*, *ywkB*(↑)

MM17	N/A	*appC*(↓), *hpr*

MM18	Lactose catabolism	*lacA*(↓), *lacR*

M8	Extracytoplasmic	*sigW*, *yceC*(↓), *yceD*(↑), *yceF*(↓), *ydjH*(↓)

M9	Cysteine biosynthesis	*cysK*(↓), *ytmI*(↓), *ytmJ*(↓), *ytmK*(↑), *ytmL*(↓), *ytnI*(↓), *ytnM*(↓), *yrzC*, *ytlI*

We found 9 modules and 18 mini-modules (MM), the latter defined as a module comprising only genes in the same operon or a simple regulon, with just a few members. Up-regulated genes are indicated by an up-arrow (↑), whereas a down-arrow (↓) indicates a down-regulated gene; genes without an arrow were not significantly detected in microarray. Physiological functions are discussed in the text. A module tagged 'N/A' means that currently not enough information exists to make a functional assignment.

#### Endospore formation and Spo0A (M2)

Our results indicate a cluster, divided into two sub-modules. The endospore formation sub-module grouped five genes participating in the formation of endospore, four of which were repressed (*citG*, *dppE*, *spoVG*, *yxnB*) and one was induced (*hag*). This data is in accordance with a previous report where AbrB was identified as repressing the aforementioned genes in a regulatory process known as catabolic repression of sporulation [14]. The second sub-module was composed of seven genes encoding for sporulation functions; six of which were induced (Table 1) with their transcription depending on SpoA and the sigma factor D (Sigma D), and one of which (Table 1) was repressed with its transcription depending on Sigma D.

#### Spore and prespore formation (M3)

In this module, we found 39 genes responding to the presence of glucose; 28 of these were repressed and the others were induced (Table 1). This cluster was subdivided into 2 sub-modules. The first one shows genes whose products are associated with pre-spore formation, germination and cell wall components [19-21]. The second sub-module is composed of 19 genes acting in the formation of spores, mainly regulated by Sigma B. With the exception of the induced genes (*csbX*, *yjgB*, *gcaD, ypuB yotK *and *spoIIQ*), all the other genes in these sub-modules were repressed when under the LB+G condition, a result consistent with the fact that genes involved with sporulation processes are repressed in the presence of non-restrictive nutritional conditions [21].

#### Hexuronte metabolisms (M4)

This module has genes involved in hexuronate metabolism [22], organized into two independent operons. Both operons are known to be negatively regulated by CcpA, whereas the *uxaC-yjmBCD-uxuA-yjmF-exuTR-uxaBA *operon is additionally, negatively regulated by ExuR [22]. The microarray data indicated that the genes were repressed, suggesting that CcpA represses them, when glucose is present.

#### Nitrogen metabolism and Spore coat formation (M5)

This module includes 39 genes and was divided into two sub-modules, each having related functions. The first set of four genes encode proteins that participate in nitrogen metabolism, co-regulated by the nitrogen utilization protein TnrA [23]. The second sub-module comprises 35 genes involved in the spore coat formation. A unique property of this sub-module is that all genes are regulated by the protein Sigma K, encoded by the genes *spoIIIC *and *spoIVCB *[24,25]. As all the genes belonging to this sub-module were shown to be repressed, this indicates that the sporulation regulatory program is governed by a hierarchical cascade, consisting of the transcription factors: Sigma E, Sigma K, GerE, GerR, and SpoIIID. This observed response is in accordance with previous reports [21]

#### SOS and prospore formation (M6)

Is constituted by 14 genes (Table 1) and the clustering method divided the module into two functionally defined sub-modules. The SOS sub-module possesses three genes regulated by LexA, which participate in DNA repair [26]. We found a second subunit, comprising 10 genes, regulated by Sigma E, which is the earliest-acting factor, specific to the mother-cell line of gene expression on the cascade forming the prospore [21]. As is evident in Table 1, 12 of the 14 genes participating in the cluster appear to be repressed.

As previously mentioned there are two mini-modules (MM) embedded within the giant component. The first one (MM1, Table 1), possesses the genes which encode for Sigma X and Spo0A TFs and which are involved in the sporulation process. The second mini-module (MM2 Table 1) has genes relating to glycerophospholipid metabolism that are entirely regulated by PhoP.

We found several mini-modules and two modules, separated from the giant component. The existence of these topological structures is likely to be a consequence of the fact that knowledge of the network is incomplete, the absence of genes or because certain TFs are not included in the sub-network or because of the existence of other regulatory structures, such as antiterminators, terminators and regulatory RNAs which are not considered in the network construction. For these reasons, some very well studied functions (see Table 1) such as glycolysis (MM3), respiratory function control by FNR (MM4), peroxide stress (MM5), the PTS system dependent on glucose (MM7), competence regulated by ComK (M7), the cystein module (M8) and a topological structure dependent on the sigma factor W (M9) were excluded from the giant component.

### Comparison of the glucose responsive networks found in *E. coli *and *B. subtilis*

The structure of complex transcriptional regulatory networks has been studied extensively in certain model organisms. However, understanding is still limited concerning the evolutionary dynamics of these networks in different organisms, which would surely reveal important principles of adaptive regulatory changes. The problem is more challenging when the aim is to carry out a detailed comparison of the regulatory networks of phylogenetically distant organisms. Previous works have studied the regulatory networks of *E. coli *and *B. subtilis *and assessed the conservation in their TFs and regulated genes, in the context of a broad array of sequenced genomes [27,28]. Both works make it clear that the set of regulatory genes - even global transcription factors - vary considerably from one group of organisms to another. This overview has to be significantly adjusted when closely related species are compared [29,30], where there is greater conservation between the TFs and the regulated genes. In this work, we compared the regulatory networks derived from significant transcript levels of *E. coli *and *B. subtilis *observed in a microarray experiment, assessing response to the presence of glucose. For this purpose, we took the *E. coli *sub-network previously published by our group [13] along with the one generated in this work. The *E. coli *sub-network was constructed from 380 genes and 47 TFs, listed in the RegulonDB database [31]. The comparison was carried out at 2 levels: the first one considered the conservation of orthologous genes in both sub-networks and the second took into account the modular structures of *B. subtilis *as described in this report as well as that previously published by Gutierrez-Rios *et al *[13], describing *E. coli*.

#### Identification and analysis of the orthologous genes in both *E. coli *and *B. subtilis *which respond to glucose

**Figure 3:**
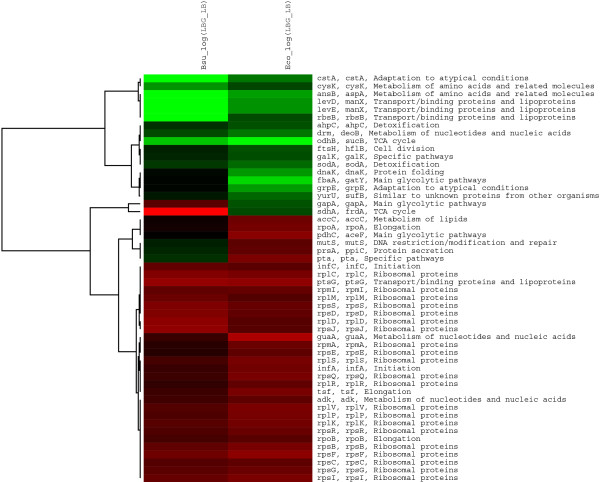
**Comparison of the significantly induced orrepressed orthologous genes in *E. coli *and *B. subtilis***. The figure illustrates a cluster of orthologous genes, comparing *B subtilis *(column 1) and *E. coli *(column 2) transcribed levels, as they respond to glucose. Induced genes are represented in red and repressed genes are represented in green. Gene names and functional class are indicated on the right hand side.

We performed a computational search for the bidirectional best hits (BBHs) found in all open reading frames for the genomes of *E. coli *and *B. subtilis*, as described in the methods section. As a result, 1199 orthologous genes were shown to be present in these two organisms. From this set, 134 genes manifested significant differences in terms of repression/activation when *B. subtilis *was grown in the presence or absence of glucose. Out of these, 52 genes were orthologous and responsive to the presence of glucose in the case of both organisms. Figure 3, shows that 47 genes exhibited the same expression pattern in the case of both organisms and five differed. These five genes are *pta *(phosphoacetyltransferase), *gapA *(glyceraldehide-3-phosphate dehydrogenase), *prsA *(peptidyl-prolyl-cis-trans-isomerase), *sdhA *(succinate deshydrogenase and *mutS *(methyl-directed mismatch repair). The *pta *gene was found to be repressed in the *B. subtilis *microarray data, a result which was inconsistent with a previous report by Presecan-Siedel *et a*l [32], which demonstrated that *pta*, as is the case with other genes involved in acetate production are induced in the presence of glucose. An induction was also observed for the *pta *gene of *E. coli *[33]. The *gapA *gene was induced in *B. subtilis *and repressed in *E. coli*. The observation was consistent with other reports where the *gapA *of *B. subtilis *and other bacillus was described as being induced in the presence of glucose, as a result of its participation in the glycolitic pathway [33]. The opposite response for *gapA *in *E. coli *may be a consequence of its participation in gluconegenesis [13]. Very little is known about the regulation of *mutS *in *E. coli *and *B. subtilis*. This gene has been described as a DNA repair protein in the context of both bacteria [34]. Something similar happens to *psrA *in *B subtilis*, also known as *ppiC *in *E. coli*; where both enzymes function as molecular chaperones. It has been reported that *prsA *is essential for the stability of secreted proteins at certain stages, following translocation across the membrane [35]. Finally, the results observed for the genes *sdhA *(succinate deshydrogenase en *B. subtilis*) and *frdA *(fumarate reductase in *E. coli*) are quite interesting. Apparently, the functions of these two enzymes seem to be different; the succinate dehydrogenases of aerobic bacteria catalyze the oxidation of succinate by respiratory quinones (succinate:quinone reductase), and the quinols are reoxidized by O_2 _(succinate oxidase) [36]. In the case of *B. subtilis*; for some time it was thought that this enzyme has only this function, but in a recent report, the authors demonstrated that resting cells are able to catalyze fumarate reduction, with glucose or glycerol. The enzymatic system for fumarate reduction in *B. subtilis *was shown to be an electron transport chain, comprising a NADH dehydrogenase, menaquinone and succinate dehydrogenase [36]. Therefore, this enzyme is able to modify its function depending on the growth condition and energetic state of the cell.

Figure 3 presents a set of genes shared by both bacteria that in addition to being orthologous display similar expression patters. Twenty of these are ribosomal genes, induced by the presence of glucose. Another seven genes are involved in the synthesis of macromolecules and a further 14 belong to cellular anabolism and catabolism of carbohydrates as well as central intermediary metabolism. Five of these are related to protective functions, four are classified as transporters and one gene encodes a protein, related to cell division.

The comparison between orthologous genes, differentially expressed in LB+G *vs *LB reveals a very small set of genes, common to both organisms. This correlates well with other works [27,28] that attribute this result to the great phylogenetic distance between these organisms. We also think this is a consequence of the small number of genes in the microarray data, shown to be differentially expressed. It is important to note that the categories conserved between these bacteria are confined to global house keeping genes, with functions associated with transcription, translation, and replication. It is also interesting to note that enzymes relating to central metabolism and energy production are also consereved and display the same behavior, whether active or inactive. The gene *sdhA *provides us with an interesting example of how orthologous genes can adapt their products to become enzymes with multiple functions, depending on their context. It would be interesting to analyze whether the regulatory response of this set of orthologous genes in other organisms preserved their original functions or adapted to alternative metabolic pathways. Hernández-Montes *et al *made an interesting contribution to this subject in terms of orthologous amino acid biosynthetic networks, where they identified alternative branches and routes, reflecting the adoption of specific amino acid biosynthetic strategies by taxa, relating their findings to differences in the life-styles of each organism [37].

Considering the 52 orthologous genes previously described, we were also interested to discover how many of the TFs regulating these were also orthologous. In Additional File 2 (see Table 2aSM) we present the orthologous expressed genes for both sub-networks, which manifest a regulatory interaction. The sub-network is composed of 43 TFs in *E. coli *and 44 in *B. subtilis *(including sigma factors). Out of these, 10 *E. coli *regulatory genes (*araC, crp, cytR, dcuR, mlc, dnaA, fur, glpR, lexA, nagC, narL*) have an orthologous regulatory counterpart in *B. subtilis *and nine *B. subtilis *regulatory genes (*ccpA, fnr, glnR, glpP, kipR, sigL, xylR, yrzC*), yufM) have one in *E. coli *(see Additional File 2: Table 3SM). As both *E. coli *and *B. subtilis *were exposed to rich media in either the presence or absence of glucose, the comparison between CcpA and CRP is especially relevant. CcpA belongs to the LacI/GalR family of transcriptional repressors [38] and CRP to the AraC/XylS family of transcription factors [39]. Both TFs fulfil the function of increasing and decreasing the activity of genes, subject to catabolic repression. The mechanism for sensing the presence or absence of glucose in both bacteria depends on the PTS system. In *B. subtilis*, PTS mediates phosphorylation of the regulatory protein HprK that in the presence of fructose 1-6 biphospate promotes the binding of CcpA to CRE sites [8]. In *E. coli*, the phosphorylation events end with the production of cyclic AMP molecules that directly activate the catabolic repression protein CRP that usually induces their regulated genes. Our results reveal that both proteins, in spite of not being orthologous and belonging to different protein families, coordinate the expression of several orthologous genes (see Additional File 2: Tables 2aSM and 2bSM). Four genes responded to glucose in both organisms and 14 in *B. subtilis*. This result may be explained, taking into account the fact that many interactions relating to every gene in the network have still not been discovered and it is also probable that the degree of sensitivity in the microarray analysis was not sufficient to detect every significant signal.

Our analysis revealed other expressed genes regulated by non-orthologous TFs that manifest similar functions. These consist of the cases of FruR (*E. coli*) and CcgR (*B. subtilis*), controlling the central intermediary metabolism, as well as RbsR (*E. coli*) and AbrB (*B*. *subtilis*), repressing genes in the presence of ribose. For instance, the AbrB, evolved to respond to additional stimulus, extending the number of elements of the regulon to sporulating functions. Finally, our results indicated that the SOS regulon control on the part of the orthologous TF LexA was not conserved [26]. The examples described previously are consistent with other findings indicating that the conservation between regulatory networks of distant organisms is in fact limited., Arguments treating this subject are directed towards the possibility of genetic duplication [40] and the adaptation of each organism to particular media [27,28], also promoting the concept that proteins evolved and took on new functions.

#### Comparison of topological units of the sub-networks between *E. coli *and *B. subtilis*

There is convincing evidence to suggest that gene duplication is a major force explaining the growth of TRNs [27,28,40]. It is possible that this modifying process affects the connectivity distribution of these networks, as has been observed in other biological networks [27]. In view of these findings, we compared the modular structures found in *E. coli *and *B. subtilis*, in order to evaluate the conservation of topological structures.

**Figure 4:**
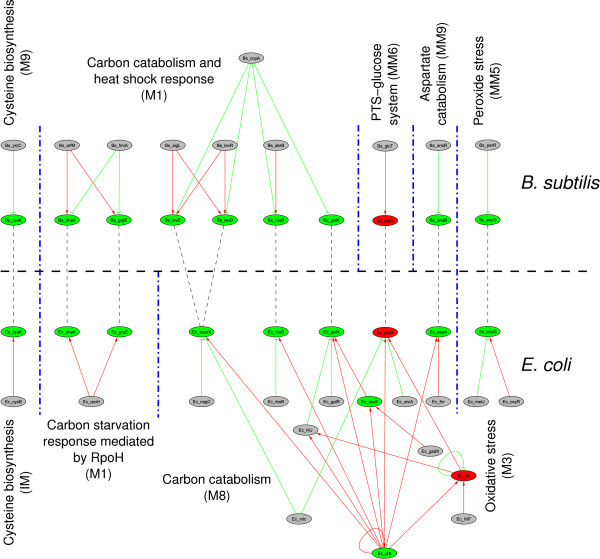
**Conserved glucose responding modules between *B. subtilis *and *E. coli***. Whereas there is extensive rewiring in the regulatory network, some modules have conserved their physiological functions and expression profile, showing the high plasticity of regulatory networks in terms of evolution. Dashed thin lines show orthology relations, whereas blue dash-dot lines bound modules. Green ellipses indicate repressed genes; red ones show activated genes and grey ones indicate genes, which are not significantly expressed. *E. coli *modules IDs are taken from Gutierrez-Rios *et al*. [13]. Regarding the aspartate catabolism module, it has been suggested that L-aspartase encoded by ansB is an strictly catabolic enzime (catalyzing the reaction aspartate → fumarate + NH_4_^+^), thus providing carbon skeletons to Krebs cycle.

A comparison was carried out, considering the modular structure of the sub-network of *E. coli *in the presence of glucose [13] and the modular structure for *B. subtilis*, generated during this study. Figure 4 presents orthologous genes that were organized into modular structures. At this level, we could see that most of the genes clustering in modules in both sub-networks, related to carbon metabolism. Those genes encoding for proteins of the PTS system were outstanding (*levDE*, *ptsG*), the degradative enzyme *galK *and the gene *rbsB *encoding as a transporter. All of the genes previously described except *ptsG *belong to the modules classified as Carbon Modules in both sub-networks. In the case of *E. coli*, genes in this module were clustered because they were regulated by CRP and in the case of *B. subtilis *by the relationship of the genes to the regulatory protein CcpA. The disconnection of *ptsG *from the carbon module in *B. subtilis *can be explained by the absence of regulation by CcpA (Figure 4, Table 1).

In both arrays, we found repression of genes encoding chaperons. Two of these, (*dnaK *and *grpE*) in *B. subtilis *are orthologous to genes in *E. coli*. In *B. subtilis*, the two orthologous and other chaperons were grouped into a sub-module with two major functions: the first one related to respiration and the second one involved in heat shock response. The regulatory protein ArfM connects all the genes in the network and HrcA controls genes related to both conditions and HrcA also controls the genes responding to heat shock. In the case of *E. coli *the genes are clearly organized into a module that includes only the heat shock genes, the organization of the module depends on the sigma factor RpoH.

We also found that respiratory functions were clustered into two groups, in the case of *B. subtilis*. The first one embedded in the sub-module concentrates anaerobic respiration and some heat shock proteins. The second set of respiratory clustered genes are also related to anaerobic functions, but in this instance they are regulated by the transcription factor FNR which is orthologous to CRP in *E. coli*. In contrast, respiratory functions in *E. coli *are clustered into one module containing proteins that control aerobic and anaerobic growth. One of the TFs in *E. coli *is FNR, for which there is no orthologous gene in *B. subtilis*. It is interesting to note, that despite not being orthologous, FNR regulates the expression of the orthologous operon *narGHJI *which encodes for all the subunits of the nitrate reductase enzyme [41,42], *narK-fnr*, where *narK *encodes a protein with nitrite extrusion activity [41,43] and the regulatory gene *fnr*. The microarray data also revealed ten genes in *B. subtilis*, known to participate in respiratory functions, where no regulatory interactions have been described (membrane bioenergetics electron transport chain and ATP synthase, see Additional File 1: Table 1SM). We also observed a pair of module clustering genes that control stress by peroxides; for *B. subtilis*, the regulatory protein PerR, whereas for *E. coli*, it is OxyR. The module shares an orthologous gene *ahpC *that was repressed in both micro arrays.

Finally, the topological arrangement, which resulted from the clustering method applied, revealed two very important differences. The first one was the case of modules related to sporulation. These were not expected to be found in *E. coli*, but occupy more than 50% of the regulatory sub-network in *B subtilis*. This finding is also not a surprise considering that sporulation is the best-studied mechanism in this organism. It is also important to mention that 74% of the genes that cluster in the sporulation modules are repressed and the genes that appeared induced in the cluster are mainly dedicated to functions such as cell wall formation, motility, ribosomal proteins, DNA replication and others not assigned to a specific class. This finding reflects the physiological importance of sporulation in this organism, which is one of the most interesting features of certain soil bacteria. It is well known that in response to nutrient limitation, *B. subtilis *cells undergo a series of morphological and genetic changes that culminate with the formation of endospores. Conversely, the presence of sufficient metabolizable carbon sources, *e. g*., glucose inhibits the synthesis of extracellular and catabolic enzymes, TCA cycle enzymes and the initiation of sporulation. This is the second difference concerning the topological arrangement of our studied organisms and a characteristic not shared by *E. coli*, which has a different life style. It would be interesting to ascertain whether in a different growth condition, the topological analysis of alternative sub-networks would manifest the same result.

## Conclusion

The analysis of transcriptome data collected under conditions of both glucose sufficiency and deficiency in a complex medium enabled us to identify functions involved in the adaptation of *B. subtilis *to these growth conditions. The known repressive effect of glucose on alternative carbon source import and metabolism were clearly demonstrated. We also were able to observe an inductive effect on the glycolitic pathway and the repressive effect on the genes related to the sporulation cascade.

A topological analysis revealed modules that include gene encoding functions, with similar physiological roles.

In a previous work, we performed a similar study under the same conditions on the Gram negative bacteria *E. coli *[13]. Analysis of orthology and topological structures, exposed coincidences in the genes that can be considered as the basic machinery of these organisms, such as replication, transcription, translation, central intermediary metabolism and respiratory functions. An outstanding discovery consisted in the fact that both bacteria manifest a similar response concerning the gene encoding chaperones, when responding to heat shock, even when these are controlled by different transcription factors (the heat shock sigma factor -Sigma H- in *E. coli *and the regulatory protein ArfM in *B. subtilis*). Also noteworthy was the identification of modules in *E. coli *and *B. subtilis*, including genes related to alternate carbon source utilization, which respond to the presence of glucose and are regulated by CRP and CcpA respectively, employing different mechanisms. Other examples were described in the results and discussion section, showing that for similar transcriptional responses, different regulatory strategies were implemented in the case of each organism. The considerable differences between the mechanism controlling gene expression and the small set of orthologous genes found in the conditions tested, are a consequence of the large phylogentic distance between these bacteria.

These analyzes also revealed how incomplete our knowledge still is, concerning gene regulation in *B. subtilis*. We are aware that processes such as catabolic repression, nitrogen assimilation and sporulation have been extensively analyzed, whereas other functions shared with *E. coli*, such as certain genes of the main glycolytic pathways, TCA cycle, and respiratory function, are not well understood. Integrative analysis of transcriptome and transcriptional regulatory data as undertaken here, as well as the comparison between organisms should provide a framework for the future generation of models. These will help explain the cell's capacity to respond to a changing environment and increase understanding of the evolutionary forces, which enable life forms to harmonize their regulatory processes in order to improve their adaptation.

## Methods

### Data analysis and identification of differential transcribed genes

Transcriptome data was obtained from previously described experiments performed with *B. subtilis *strain ST100 broth, containing 50 mM potassium phosphate, pH 7.4, and 0.2 mM L-cysteine with (LB+G) or without (LB) 0.4% glucose. The average expression data from three repeated experiments was collected from web http://biology.ucsd.edu/~msaier/regulation2/ of the *B. subtilis *antisense. DNA arrays used in this work were custom designed and manufactured by Affymetrix (Santa Clara, CA) [8].

As we only had access to the average of the crude expression data, we applied the rank product method [44]. This method is based on the calculation of rank products, from which significance thresholds can be extracted, in order to distinguish significantly regulated genes. In the case of our data, we chose a RP-value of 3.5 × 10^-2 ^as a cutoff point, and in this way we distinguished the most significant 150 up-regulated and 150 down-regulated genes. However, as we also were interested in the differential expression under both conditions, we picked up those genes exhibiting a > 3-fold change between LB and LB+G. Finally, we took the logical union of such populations. Using this method a set of 503 genes were taken into account for subsequent analysis.

As in our previous work, concerning differentially expressed genes of *E. coli *[13], the terms "induced" and "repressed" were used in this work to indicate increased or decreased transcript levels, respectively. These terms do not imply a particular mechanism for gene regulation.

### Extraction of condition-specific sub-networks

For each microarray condition LB+G/LB, we reconstructed a condition specific sub-network as follows. From the transcriptional regulatory network of *B. subtilis*, we extracted the significant genes identified in the microarray condition, the TFs regulating their expression, and the transcriptional interactions between TFs and their regulated genes. In these sub-networks, nodes represent genes and edges represent the transcriptional interactions. Known regulatory sites and transcriptional unit organization were obtained from DBTBS [45].

### Identification of condition-specific modules

We identified the LB+G/LB condition-specific modules applying to the condition specific sub-network, the methodology described in Resendis-Antonio *et al *[46] and Gutierrez-Rios *et al *[13]. Specifically, we clustered the genes based on their shortest distance within the network. Afterwards, we annotated each gene with its corresponding microarray expression level. The dendogram generated by the clustering algorithm was decomposed into modules and sub-modules. Hierarchical clustering algorithms produce a dendogram by iteratively joined pairs of data, with the closest correlation levels. We analyzed the distribution of correlation values, observing that ~90% (228 from 254) of the nodes in the dendogram have a correlation value greater than 80%. Hence, in order to isolate modules, we pruned every node with a correlation of less than 80% from the dendogram. In addition, to identifying sub-modules, we then pruned the dendogram once again; this time removing all the nodes with a correlation of less than 90%.

### Detection of orthologous genes

A simple method for predicting the orthologous proteins present in two organisms is to search for a pair of sequences, Xa in organism Ga and Xb in organism Gb, such that a search of the proteome of Gb with Xa indicates Xb to be the best hit. We made this comparison using the Blastp program [47,48] with the *E. coli *and the *B subtilis *genome as input. If the protein in each genome has the highest E-value and an upper threshold of 10^-5 ^in both genomes, we considered them to be orthologous. From this set we selected the significant expressed genes, published in our previous work run under the same conditions of LB growth, in the presence or absence of glucose [13].

### Clustering of microarray data of orthologous genes

We applied a hierarchical centroid linkage clustering algorithm [49,50] to the log ratios of the differences between the orthologous genes of *E. coli *and *B. subtilis*, with the correlation un-centered as a similarity measure... The clustering results were visualized using the Treeview program [51].

## List of abbreviations

CRE, SM, LB, LB+G, TF, PTS, *B. subtilis*, *E. coli*.

## Authors' contributions

CDV contributed with construction of the regulatory network, microarray and module analysis. JAF-G contributed with the discussion for the selection of microarray data, performed the construction of topological modules and comparison of modular subunits. GG contributed with the analysis and interpretation of microarray data for the physiological sections. RMG-R contributed to the analysis and interpretation of the microarray data in terms of the regulatory network, elaboration of programs for data management as well as a discussion concerning the selection and processing of microarray. All authors wrote, read and approved the final manuscript.

## Supplementary Material

Additional File 1 **Supplementary Table 1SM**. "VazquezHernandezSupplementary-Material_1" and contains tables from 1 to 3, describe in the manuscript as Table 1SM. Click here for file

Additional File 2 **Supplementary Tables 2-3SM**. "VazquezHernandezSupplementary-Material_2" and contains Tables 2 to 3, described in the manuscript as Table 2aSM, Table 2bSM, and Table 3SM. Click here for file
