# Supplementary material for: Identification of network topological units coordinating the global expression response to glucose in Bacillus subtilis and its comparison to Escherichia coli
Source: BMC Microbiol. 2009 Aug 24;9:176. doi: 10.1186/1471-2180-9-176 (PMC2749860; doi:10.1186/1471-2180-9-176)
Supplement: Additional File 2 — Supplementary Tables 2-3SM. "VazquezHernandezSupplementary-Material_2" and contains Tables 2 to 3, described in the manuscript as Table 2aSM, Table 2bSM, and Table 3SM. [file 1471-2180-9-176-S2.pdf]

**Gene *B. subtilis***	**Regulatory Phrase**	**Gene *E. coli***	**Regulatory Phrase**
ahpC	PerR(-) sigma A	*ahpC*	MetJ(-) OxyR(+) sigma70
ansB	AnsR(-) sigma A	*aspA*	CRP(+) FNR(+) sigma70
cstA	unknown	*cstA*	CRP (+)
cysK	YrzC(-) sigmaA	*cysK*	CysB (+) sigma70
dnaK	ArfM(+) HrcA(-) sigmaA	*dnaK*	sigma32
drm	sigma A	*deoB*	CRP(+-) CytR(-) DeoR(-) Fis(+) sigma70
fbaA	unknown	*fbaA*	CRP (+) FruR (-)
ftsH	sigma A	*hflB*	sigma32
galK	CcpA(-)	*galK*	CRP(+-) GalR(-) GalR(-) HNS(-) HU(-) sigma70
gapA	CggR(-) sigA	*gapA*	sigma32
grpE	ArfM(+) HrcA(-) sigma A	*grpE*	sigma32
guaA	sigma A	*guaA*	CRP (+) DnaA(-) Fis(+) PurR(-) sigma70
levD	CcpA(-) LevR(+) sigma L	*manX*	CRP(+) Mlc(-) NagC(-) sigma70
levE	CcpA(-) LevR(+) sigma L	*manX*	CRP(+) Mlc(-) NagC(-) sigma70
infA	RplT (-) sigmaA	*infA*	unknown
infC	RplT (-) sigmaA	*infC*	unknown
odhB	sigma A	*sucB*	ArcA (+-) FNR(-) IHF(-

			) CRP(+) sigma70
pdhC	sigma A	*aceF*	CRP(+) FNR(+-) PdhR(-) sigma70
pta	CcpA(-) sigma A	*Pta*	unknown
ptsG	GlcT(+) sigma A	*ptsG*	ArcA(-) CRP(+) DgsA(-) Mlc(-) SoxS(-) sigma70
sdhA	sigma A	*frdA*	DcuR(+) FNR(+) NarL(-) sigma70
rbsB	AbrB(+) CcpA(-) sigma A	*rbsB*	CRP (+) RbsB (-) sigma70
rplC	RplT (-) sigmaA	*rplC*	ArcA (-) FNR (+)
rplD	unknown	*rplD*	ArcA (-) FNR (+)
rplM	RplT (-) sigmaA	*rplM*	FNR (-)
rplP	unknown	*rplP*	ArcA (-) FNR (+)
rplR	RplT (-) sigmaA	*rplR*	unknown
rplS	RplT (-) sigmaA	*rplS*	FNR (-)
rplV	unknown	*rplV*	ArcA (-) FNR (+)
rpmI	RplT (-) sigmaA	*rpmI*	unknown
rpoB	sigma A	*rpoB*	unknown
rpsD	sigma A	*rpsD*	sigma70
rpsF	ComK(+)	*rpsF*	unknown
rpsJ	unknown	*rpsJ*	ArcA (-) FNR (+)
rpsQ	unknown	*rpsQ*	ArcA (-) FNR (+)
rpsR	ComK(+)	*rpsR*	ArcA (-) FNR (+)
sodA	unknown	*Soda*	ArcA (-) CRP (+) FNR (-) Fur (-) IHF (-) Rob (+) SoxS (+)
yurU	unknown	*sufF*	Fur (-) IHF (+) IscR (+)

			OxyR (+)

Table S2a. Orthologous genes with a described regulatory interaction and a significant level of expression in both organisms. Columns 1 and 3 shows affected orthologous genes in both transcriptomes, repressed genes are color in green and induced genes in red. Columns 2 and 4 exhibit the proteins regulating each gene and their functions as repressors (-), activators (+), or dual regulators over each gene are also shown.

.

Gene B. subtilis	Regulatory Phrase	Gene E. coli	Regulatory Phrase
acoR	CcpA(-) sigmaG	ygeV	unknown
adhB	sigmaG	ybdR	unknown
ahpF	PerR(-) sigma A	ahpF	MetJ (-) OxyR (+) sigma70
appC	Hpr(-)	gsiD	ArcA (+) FNR (-)
araB	AraR(-) CcpA(-) sigmaA	araB	AraC (+ -) Crp (-) sigma70
arsR	ArsR(-) sigmaA	arsR	ArsR (-) sigma70
bglH	CcpA(-) LicT(+) sigmaA	bglB	CRP (+) FIS (-)
bglP	CcpA(-) LicT(+) sigmaA	bglF	CRP (+) FIS (-)
clpP	CtsR(-) sigmaA sigmaB	clpP	sigma32 sigma70
comGB	ComK(+) sigmaA	gspF	HNS (-) sigma70
cspB	ComK(+) sigmaA	cspC	unknown
cydC	YdiH(-) sigA	cydD	ArcA (+) FNR (+) NarL (+)
dctP	CcpA(-) DctR(+) sigmaA	dctA	ArcA (-) CRP (+) DcuR (+) sigma70
eno	CggR(-) sigmaA	eno	FruR (+)
ffh	sigmaA	ffh	unknown
gcaD	sigmaA sigmaB	glmU	NagC (+-) sigma70

glnA	GlnR(-) TnrA(-) sigmaA	glnA	CRP (+-) FIS (+) NtrC (+-) sigma54 sigma70
glpK	CcpA(-) GlpP(+) sigmaA	glpK	CRP (+) GlpR (-) sigma70
glpQ	GlpP(+) PhoP(+) sigmaA	glpQ	CRP (+) FNR (+) FIS (+) GlpR (-) IHF (-) sigma70
glpT	GlpP(+) PhoP(+) sigmaA	glpT	sigma70
groEL	HrcA(-)	groL	sigma70,sigma32
groES	HrcA(-)	groS	sigma70,sigma32
gutB	GutR(+) sigmaA	ydjJ	unknown
gyrA	sigmaA	gyrA	CRP (+) CspA (+) FIS (-) sigma70
hag	CodY(-) sigmaD	fliC	GadE (+) HNS (+) sigma28
Icd	CcpA(-) CcpC(-) sigmaA	icd	ArcA (-) FruR (+) sigma70
idh	CcpA(-) IolR(-) sigmaA	yceM	unknown
iolC	CcpA(-) IolR(-) sigmaA	kdgK	unknown
licA	CcpA(-) LicR(+) sigmaA	chbA	CRP (+) ChbR (+-) NagC (-)

licB	CcpA(-) LicR(+) sigmaA	chbB	CRP (+) ChbR (+-) NagC (-)
licH	CcpA(-) LicR(+) sigmaA	chbF	unknown
menB	sigmaA	menB	sigma70
mmgA	CcpA(-) sigmaE	yqeF	unknown
narG	Fnr(+) sigmaA	narG	FNR (+) FIS (+) IHF (+) NarL (+) RstA (+) sigma70
narH	Fnr(+) sigmaA	narH	FNR (+) FIS (+) IHF (+) NarL (+) RstA (+) sigma70
odhA	sigmaA	sucA	ArcA (+-) CRP (+) FNR (-) Fur (+) IHF (-) sigma70
pdhD	sigmaA	lpd	ArcA (-) CRP (-) FIS (+) PdhR (-) sigma70
pgk	CggR(-) sigmaA	pgk	CRP (+) FruR (-) sigma70
pgm	CggR(-) sigmaA	gpmM	unknown
ptsI	GlcT(+) sigmaA	ptsI	CRP(+-) DgsA (-) FruR (+-) sigma70
purQ	PurR(-) sigmaA	purL	PurR (-) sigma70
rbsA	AbrB(+) CcpA(-)	rbsA	CRP (+) RbsB (-)

	sigmaA		sigma70
rbsC	AbrB(+) CcpA(-) sigmaA	rbsC	CRP (+) RbsB (-) sigma70
rbsD	AbrB(+) CcpA(-) sigmaA	rbsD	CRP (+) RbsB (-) sigma70
rbsK	AbrB(+) CcpA(-) sigmaA	rbsK	CRP (+) RbsB (-) sigma70
sdhB	sigmaA	frdB	ArcA (-) DcuR (+) NarL (-) sigma70
spsI	SpoIVCB(+) SpoIVCB(+)	rffH	unknown
ssb	ComK(+)	ssb	ArcA(-) LexA (-) sigma70
ssuC	YrzC(-) sigmaA	ssuC	Cbl (+) CysB (-) FNR (+) IHF (+-) sigma70
tpiA	CggR(-) sigmaA	tpiA	unknown
treA	CcpA(-) TreR(-) sigmaA	treC	ArcA (+) CRP (+) TreR (-) sigma70
treP	CcpA(-) TreR(-) sigmaA	treB	ArcA (+) CRP (+) TreR (-) sigma71
xylA	CcpA(-) CcpB(-) XylR(-) sigmaA	xylA	CRP (+) XylR (+)
xynP	CcpA(-) XylR(-) sigmaA	yagG	unknown

ycsF	KipR(-) SpoIIIC(+)<br><br>SpoIVCB(+)<br><br>TnrA(+)	ybgL	unknown
yisZ	SpoIIIC(+)<br><br>SpoIVCB(+)	cysC	CysB (+) sigma70
yjmC	CcpA(-) ExuR(-)<br><br>sigmaA sigmaE	allD	AllS (+)
yncD	sigmaE	alr	unknown
yngK	SpoIIIC(+)<br><br>SpoIVCB(+)	yddW	unknown
ywkB	YufM(+) sigmaA	yfdV	unknown

Table S2b. Orthologous genes with a described regulatory interaction but only significant expressed in *B. subtilis.*

Column 1 exhibit affected orthologous genes in both *B. subtilis*, repressed genes are color in green and induced genes in red. Column 3 shows orthologous genes without a change in their level of expression in *E. coli*. Columns 2 and 4 put on view the proteins regulating each gene and their functions as repressors (-), activators (+), or dual regulators over each gene are also shown.

Regulatory gene *B.subtilis*	Description	Regulatory gene *E. coli*	Description
ccpA	Carbon catabolite control protein involved in glucose regulation of several genes; mediates carbon catabolite repression (CCR)	cytR	Regulator for deo operon, udp, cdd, tsx, nupC, and nupG
dnaA	initiation of chromosome replication (DNA	dnaA	Putative regulator; DNA - replication, repair,

	synthesis)		restriction/modification
Fnr	regulation of anaerobic genes (narK-fnr, narGHJI, lctEP, alsSD)	crp	Catabolic repressor protein
Fur	Negative regulation of siderophore biosynthesis and transcription of ferri-siderophore uptake genes	fur	Iron regulatory gene
glcK	Glucose kinase	dgsA	Regulator of enzymes phosphotransferase (PTS) and phosphoenolpyruvate (PEP) systems
glcR	Negative regulation of the phosphotransferase system	glpR	regulator; Energy metabolism, carbon: Anaerobic respiration
glnR	Regulates gene expression in response to changes in nitrogen availability	mlrA	MlrA is a regulator of curli production i
glpP	Transcription antiterminator	ygcP	predicted anti-terminator regulatory protein
Hbs	Non-specific DNA-binding protein HBsu; signal recognition particle-like (SRP) component	hupA	Basic proteins - synthesis, modification
kipR	Regulator of the histidin kinase operon	yagI	CP4-6 prophage; predicted DNA-binding transcriptional regulator
lexA	Negative regulation of the SOS regulon (DNA-damage inducible genes)	lexA	Negative regulation of the SOS regulon (DNA-damage inducible genes)
sigL	RNA polymerase sigma-54 factor (sigma-L)	rpoN	RNA polymerase, sigma 54 (sigma N) factor
xylR	Negative regulation of the xylose operon (xylAB)	nagC	The NagC, "N-acetylglucosamine," transcriptional dual regulator participates in regulating the phosphotransferase system (PTS). Its function is to coordinate the biosynthesis of the amino sugars, D-glucosamine (GlcN) and N-acetylglucosamine (GlcNAc) with their catabolism

ybfI	unknown	araC	regulates genes involved in arabinose catabolism and transport
ybfP	unknown	marA	**m**ultiple **a**ntibiotic **r**esistance," is a transcriptional repressor that controls genes involved in multiple antibiotic resistance and susceptibility
yclJ	unknown	ompR	Control the osmoregulated biosynthesis of the porin proteins OmpF and OmpC
yhcZ	unknown	narL	controls the expression of several genes involved in anaerobic respiration and fermentation.
yrzC	Repressor of cysteine biosynthesis operons	iscR	IscR (iron-sulfur cluster regulator)
yufM	unknown	dcuR	Putative regulator; Not classified

Table S3. Orthologous regulatory proteins and their regulatory functions in *B. subtilis* and *E. coli.*
